# Supplementary material for: Noise Indicators Relating to Non-Auditory Health Effects in Children—A Systematic Literature Review
Source: Int J Environ Res Public Health. 2022 Nov 24;19(23):15633. doi: 10.3390/ijerph192315633 (PMC9739374; doi:10.3390/ijerph192315633)
Supplement: Supplementary file 1 [file ijerph-19-15633-s001.zip › Supplementary_Material.pdf]

Supplementary Materials: Noise Indicators Relating to Non-Auditory Health Effects in Children - A Systematic Literature Review

Michail Evangelos Terzakis, Maud Dohmen, Irene van Kamp, and Maarten Hornikx

1. Searching Query

The search query was defined such,

{Noise Indicators} *AND* {Health Effects} *AND* {Children} $\Longrightarrow$ {"noise indicator" *OR* "noise indicators" *OR* "noise index" *OR* "noise indices" *OR* "noise metric" *OR* "noise metrics" *OR* "noise exposure" *OR* "environmental noise" *OR* "urban noise" *OR* "industrial noise" *OR* "ambient noise" *OR* "traffic noise" *OR* "aircraft noise"} *AND* {annoyance *OR* "cognitive performance" *OR* "cognitive development" *OR* cognition *OR* "mental disorder" *OR* "mental disorders" *OR* "mental health" *OR* psychological *OR* psychophysiological *OR* rest *OR* restoration *OR* sleep *OR* stress} *AND* {neonatal *OR* neonatals *OR* fetus *OR* new-born *OR* infant *OR* infants *OR* baby *OR* babies *OR* toddler *OR* toddlers *OR* adolescent *OR* adolescents *OR* juvenile *OR* juveniles *OR* student *OR* students *OR* child *OR* children}.

2. Results per Health Outcome

Table S1. Cardiovascular-based studies. NM is referred to number of measurements, EFM to the exclusion of the first blood measurement, CS to the cuff-size, SBP and DBP to systolic and diastolic blood pressure.

Article (Participants, Age Range)	Noise Source (Assessment: Method)	Noise Assessment Location (Environment)	Noise Indicators	Health Parameter (Device)	NM	EFM	CS	Outcomes
Wallas et al. (2019)† [1] (n=2597, 16yrs)	Road Traffic Noise (Calculations: NPM)	Homes (Outdoor: Most exposed façade)	L_{den}	SBP, DPB (Omron M6)	3	N/A	Yes	SBP presented inverse associations with road traffic noise (crude model). Higher SBP association in children, whose mothers exposed to occupational noise $\geq$ 70 dB(A). DBP associations similar to SBP but weaker.
Babisch et al. (2009)† [2] (n =1048, 10-14)	Road traffic Noise (Measurements: NORSONIC Type 116)	Homes (Outdoor: In front of the child's open room window)	$L_{A,S,15min}$ (08:00 and 21:30)	SBP, DPB (Datascope Accutorr Plus)	2	N/A	N/A	Significant increase of both systolic and diastolic blood pressure.
Belojevic et al. (2008)† [3] (n=328, 3-7yrs)	Road Traffic Noise (Measurements: B&K 4426)	Home and School (Outdoor: In front of Residences and kindergarten)	$L_{A,eq,15m}$ (School:09:00-10:30, 13:30-15:00, Home: 22:00-00:00, 00:30-01:30)	SBP, DBP (Mercury sphygmomanometer: Fazzini)	2	Yes	Yes	Increase of SBP is associated to high noise residences/kindergartens. SBP is associated to noise. Non-significant difference between DBP and groups. Noise is not related to DBP.
van Kempen et al. (2010)† [4] (n=2844, 9-11yrs)	Aircraft Noise (Calculations: Noise Contours) Road Traffic Noise (Calculations: CRTN and others)	School (Outdoor: Around Airports)	$L_{A,eq,16h}$ (07:00-23:00)	SBP, DBP (OMRON 711)	3	N/A	Yes	Annoyed children presented associations between road traffic noise and diastolic blood pressure.
Lercher et al. (2013)† [5] (n=115, mean 10.1yrs)	Road Traffic Noise (Calculations: Modelled Traffic Noise)	Home (Outdoors: Child's Location)	Ldn	SBP, DBP (Bosch, Sysditon)	2	Yes	Yes	Non-significant increase between both SBP and DBP in children living in high noise areas.

†: Cross-sectional study

Table S2. Neuroendocrine-based studies. NM and Time are referred to the number and time of health-based measurements.

Article (Participants, Age Range)	Noise Source (Assessment: Method)	Noise Assessment Location (Environment)	Noise Indicators	Health Parameter (Device)	NM	Time	Outcomes
Wallas et al. (2018)‡ [6] (n=2597, 16yrs)	Road Traffic Noise (Calculations: NPM)	Homes (Outdoor: Most exposed façade)	L_{den}	Saliva cortisol (Sterille rolls: Salivette)	2	Day Evening	Road traffic noise is not related to saliva cortisol levels.
Cantuaria et al. (2018)† [7] (n=165, 5wks)	Road traffic noise (Calculations: SiRENE Model)	Homes (Outdoor: Most exposed façade)	L_{den}	Cortisol, Cortisone, α-THF, THF, α-, β-Cortol, THE, α-, β-Cortolone (GC 7890A)	1	N/A	Non-significant increase between cortisol and high levels of L_{den}. Marginal significant increase of cortisone with high levels of L_{den}. Significant increase of α-THF in relation to high levels of L_{den} (Crude/Basic Models). Significant decrease of THF in relation to high levels of L_{den}. Non-significant increase of α-Cortol in relation to high levels of L_{den} (Crude Model). Marginal significant decrease of α-Cortolone in relation to high levels of L_{den} (new-borns). Marginally significant increase of β-cortolone in relation to high levels of L_{den} (new-borns). Non-significant increase of Glucocorticoids in relation to high levels of L_{den} (Crude/Basic). Non-significant decrease of glucocorticoids in relation to high levels of L_{den} (Full Model). Non-significant decrease of THE in relation to high levels of L_{den} (new-borns). Non-significant increase of β-cortol in relation to high levels of L_{den} (Crude and basic model). Non-significant decrease of β-cortol in relation to high levels of L_{den} (Full-Model). Non-significant increase of cortisol/cortisone sum in relation to high levels of L_{den}.
Haines et al. (2001)† [8] (n=340, 8-11yrs)	Aircraft Noise (Calculations: Noise Contour and Measurements: DAT)	School (Outdoor: Selected Schools) and School (Indoor: Classroom-Performance)	SEL	"Baseline cortisol" & "time 2 cortisol" (Salivette: "Magic Cortisol" RIA kit)	2	Before, after tasks	Baseline cortisol is not related to "Time 2 cortisol". Noise exposure in school is not related to cortisol (i.e., from baseline to post-testing).
Lercher et al. (2013)† [5] (n=115, mean 10.1yrs)	Road traffic noise (Calculations: Modelled traffic noise)	Home (Outdoors: Child's Location)	L_{dn}	Free Cortisol and 20a-dihydrocortisol (HPLC)	1	8h	Non-linear increase of cortisol levels with respect to the noise exposure levels. Significant increase of the 20a-dihydocortisol in relation to high noise group.

†: Cross-sectional study, ‡: Follow-up study

Table S3. Cognitive development-based studies.

Article (Participants, Age Range)	Noise Source (Assessment: Method)	Noise Assessment Location (Environment)	Noise Indicators	Health Parameter (Method)	Outcomes
Clark et al. (2013)‡ [9] (n=461, 15.5-17.7yrs)	Aircraft Noise (Calculations: Noise Contours)	Primary and Secondary schools (Outdoor)	$L_{A,eq,16h}$ (07:00-23:00)	Reading sentence completion (SRS1-Level 3)	1dB increase of noise exposure (primary & secondary) and cumulative noise exposure is related to non-significant decrease in reading.
Klatte et al. (2017)† [10] (n=1243, 7-10yrs)	Aircraft Noise (Calculations: Noise Contours)	Schools and homes (Outdoor)	Schools: $L_{A,eq,6h}$ (08:00-14:00) Homes: $L_{A,eq,12h}$ (06:00-18:00) and $L_{A,eq,10h}$ (20:00-06:00)	Story Comprehension ("AuditoryMemory" subtest: German-"IDS; Grob, Meyer,& Hagmann-von Arx, 2009"). Reading speed & accuracy-single words, sentences, and short paragraphs: (SRC)	Aircraft noise exposure is not related to story comprehension. Full sample is related to non-significant decrease in performance w.r.t. migration background. A 10dB exposure in schools is related to decrease in global reading & word scores (1/10 of a SD), text reading (1/8 of a SD), and reading delay (1 month). Sentence Reading is not related to aircraft noise exposure. Migration background sample presented non-significant decrease in reading scores. In non-migration background sample, an increase of 10dB is related to a decrease in global & text reading (1/7 of a SD), word reading (1/6 of a SD), & reading delay (1.5 month).
Stansfeld et al. (2005)† [11] (n=2844, 9-10yrs)	Road traffic & Aircraft Noise (Calculations: UK-SP: Noise Contours/CRTN Method, NL: Modelled data + Validation Measurements)	School (Outdoor/Indoor: At time testing)	$L_{A,eq,16h}$ (07:00-23:00)	Sustained Attention (Adapted TPT for classroom use). Episodic Memory (Adapted Task from the CMS). Working Memory (modified version of search and memory task) Reading comprehension (SRS, CITO)	Sustained attention & working memory are not related to aircraft & road traffic noise. Aircraft noise exposure is significantly related to a decrease in re-cognition, whereas a non-significant decrease presented in relation to recall or conceptual recall. Road traffic noise is significantly related to increase in information & conceptual recall. Recognition memory is not related to road traffic noise. Prospective memory is not related to aircraft & road traffic noise. Significant increase in comprehension & recognition in relation to chronic aircraft noise exposure. 5dB and 20dB increase of aircraft noise exposure is associated to reading delay; UK: 2-month, NL: 1-month & NL & SP: 1/8, UK: 1/5 of a SD, respectively. Reading comprehension is not related to road traffic noise.
Seabi et al. (2015)‡ [12] (n=650/178, 9-15/10-16yrs)	Aircraft Noise (Measurements: SVAN 955)	School (Outdoor: Surrounding areas)	$L_{A,eq,2.5h}$ (08:00-10:30)	Reading comprehension (SRS2 Test)	Low noise areas' children presented non-significant increase in reading comprehension mean scores (2010 & 2011).
Haines et al. (2001)† [8] (n=340, 9-11yrs)	Aircraft Noise (Calculations: Noise Contour + Measurements: DAT)	School (Outdoor: Selected schools) and School (Indoor: Classroom-Performance)	$L_{A,eq,16h}$	Long-Term Memory (Recognition, recall of reading material). Short-Term Memory (serial digit recall task) Reading ability and reading standards (SRSL2)	Aircraft chronic exposure is not related to recall performance, short-term memory, and recognition (8-schools). Children in 4 high noise schools (7-schools) presented significant lower score in long-term memory recognition tasks. Chronic aircraft noise exposure is not related to reading comprehension (8-schools). Reading comprehension is significantly related to noise exposure and children in 3-low noise schools. Reading ability is related to 6 months delay.
Haines et al. (2001)† [13] (n=275, 9-11yrs)	Aircraft Noise (Measurements: SLM)	Home (Outdoor: Residential areas) and School (Indoor: Classroom)	$L_{A,eq,16h}$	Sustained Attention (TEA-Ch) Reading Comprehension (UK S Suffolk Reading Scale (Level 2))	Significant reduction in sustained attention (all schools) for high aircraft noise levels. After adjustment, significance remained in seven schools. Comprehension is not related to chronic aircraft noise exposure (8 schools). Significant reduction in reading comprehension for high noise 4-schools (seven school). Significant differences in performance (follow-up) between high and low noise children (eight schools) (Crude model).
Clark et al. (2006)†† [14] (n=275, 9-11yrs)	Aircraft Noise (Calculations: Models) and Road-) Traffic Noise (Measurements: SLM)	School and Home (Outdoor) and School (Outdoor: Schools' façade)	$L_{A,eq,16h}$	Sustained Attention (TEA-Ch) Reading Comprehension (NST: UK: SRSL-2, NL: CITO, SP: ECL-2)	Decrease in comprehension is significantly related to increase in aircraft noise exposure (School). An increase of 5dBA is associated to decrease in reading test performance. An increase of 20dBA is associated to decrease reading test. Aircraft noise at home is related to aircraft noise at school. Decrease in comprehension is significantly related to increase of aircraft noise exposure at Home. Comprehension is not related to chronic road traffic noise at school.
Matsui et al. (2004)† [15] (n=236, 8-9yrs)	Aircraft Noise (Calculations: Noise Contours)	Schools and Home (Outdoor: Surrounding Areas)	$L_{A,eq,16h}$ (07:00-23:00)	Sustained Attention (TEA-ch), Long-term Memory Recall & Recognition (Adapted CMS) Reading Comprehension (SRS)	Sustained attention is not related to chronic noise exposure. Dose-response relationship: Noise level are significantly related to immediate & delayed recall (Adjusted Model). Immediate & delayed recall presented significant ORs w.r.t. high noise group. Reading comprehension & delayed recognition did not present significant differences between two groups. Reading mean score, reading (difficult) trend tests & did not present dose-response relationships. ORs (other three outcomes) >1 with respect to high noise groups.
Matheson et al. (2010)† [16] (n=2844, 8.10-12.10yrs)	Aircraft & Road Traffic noise (Calculations: Noise Contours + Different Traffic Models)	Schools and Home (Outdoor: Surrounding Areas)	$L_{A,eq,16h}$	Episodic Memory (Children's Memory Scale), Prospective Memory (Shield and Dockrell test)	Chronic road traffic noise exposure is related to a significant increase in information recall. A 5dB increase is related an increase in information recall. Exposure-effects are related to road traffic noise. Chronic road traffic noise exposure is significantly related to increase in conceptual recall. A 5dB increase is related to increase in conceptual recall. Linear exposure effects are associated to road traffic noise (Adjusted Model) Chronic aircraft noise exposure is significantly associated to decrease in recognition memory. A 5dB increase is associated to decrease in recognition. Aircraft noise is related to recognition memory. Chronic road traffic noise exposure is not related to recognition memory. Prospective memory is not related to chronic aircraft and road traffic noise exposure.

†: Cross-sectional study, ‡: Follow-up study, ††: Longitudinal study

Table S4. Cognitive performance-based studies.

Article (Participants, Age Range)	Noise Source (Assessment: Method)	Noise Assessment Location (Environment)	Noise Indicators	Health Parameter (Method)	Outcomes
Xie et al. (2011)* [17] (n=n.sp., 12-13yrs)	Road-Traffic Noise (Calculations: Noise Maps Database)	School (Outdoor: At the façade)	L_S, $L_{S,max}$, $L_{S,min}$, L_S, $L_{S,10}$, $L_{S,90}$	Academic Achievements (Key Stage 4, CVA score, overall & persistent absence)	Students' persistent absence is significantly related to intrusive noise environments of road-side school group ($L_{S,10}$). Academic achievements are not related to noise exposure and therefore to noise indicators. Environmental noise levels (all secondary schools) are not related to academic achievement indicators.
Pujol et al. (2014)† [18] (n=587, 8-9yrs)	Urban Noise (Calculations: ECEND 2002/49/CE)	School and Home: (Outdoor: In front of child's bedroom and school façade).	L_d, (06:00-18:00), L_e, (18:00-22:00), L_n, (22:00-06:00), L_{den}	Mathematics Test Performance (NAS Test)	Increase of L_{den} at home is significantly related to pupils, who have already repeated a year. Mathematics scores are not associated to L_{den} at home or L_d school. Marginally significant decrease in Mathematics score in relation to L_{den} at home and L_d at school. Significant decrease in Mathematics score in relation to L_d at school (Adjusted Models). Similar results: L_d, L_e, or L_n (Home) instead of L_{den} (Home). Significant decrease in French score in relation to L_{den} at home or/and L_d at school (Crude Model). Marginally significant decrease in French score in relation to L_{den} at home and L_d at school (Adjusted Models). Significant and non-significant decrease in French score in relation to L_d at schools and L_{den} at home respectively (Further Adjusted Models). Similar results: L_d, L_e, or L_n (Home) instead of L_{den} (Home).
Van Kempen et al. (2010)† [4] (n=2884, 9-11yrs)	Aircraft Noise (Calculations: Noise Contours) and Road Traffic Noise (Calculations: NSM Methods).	School (Outdoor: Around Airports)	$L_{A,eq,16h}$ (07:00-23:00)	Coordination (Hand-eye Coordination Test) Perceptual Coding and Attention (SDST)	Significant difference between SAT and DMST, only at the difficult part. Significant increase in Faults (SAT-Switch condition) in relation to children annoyed by aircraft noise at school. Significant lower Span length (DMST) in relation to children annoyed by aircraft noise at school. Non-significant difference between road traffic annoyed and not-annoyed children in the overall scores.
Haines et al. (2002)† [19] (n=11000, 11yrs)	Aircraft Noise (Calculations: Noise Contours)	Schools (Outdoor:School Areas)	$L_{A,eq,16h}$	English, mathematics, & science (SAT: Key Stage 2)	Aircraft noise exposure is related to English mean scores (Crude Model), to reading, spelling, handwriting, & writing performance. Loss of significance after further adjustment. Increase of aircraft noise exposure is significantly associated to decrease in reading performance (Crude Model). Aircraft noise is related to mathematical performance. Increase in aircraft noise is significantly related to decrease in mathematics performance (Crude Model). Aircraft noise is not associated to science performance.
Shield et al. (2008)* [20] (n=n.sp./24, 7, 11,6-7/10-11yrs)	Ambient Noise (Measurements)	Schools (Outdoor: Most exposed façade at 4m and Indoor: Occupational, Non-occupational Classrooms, Corridors, Foye, Halls, Occupational, Non-occupational Hall, Classes: Nurs, Year 1-6)	$L_{A,eq,5m}$, $L_{A,10,5m}$, $L_{A,90,5m}$, $L_{A,max,5m}$	English, mathematics, & science (SAT: Key Stage 2)	[External] Borough A: KS1 & KS2-English test scores are significantly associated to $L_{A,90}$ (All Schools). KS2-Other Subjects' test scores are significantly associated to $L_{A,max}$. [Adjustment] FSM: $L_{A,max}$ is significantly correlated to mathematics (strongest), Science & Average score (KS2). EAL: $L_{A,max}$ is significantly associated to KS2 subjects, with Mathematics (strongest). SEN: Mathematics & Science are significantly associated to external noise and $L_{A,max}$ (strongest) (KS2). [Schools $L_{A,eq}$ >60dBA] Mathematics scores (KS1) are significantly correlated to $L_{A,90}$ (All Models). An increase of 10dBA w.r.t. $L_{A,eq}$, $L_{A,max}$, $L_{A,90}$ is associated to a decrease in KS1 & KS2-required Levels. Borough B & C: In all schools, test scores are not significantly related to external noise. [Schools $L_{A,eq}$ >60dBA] SAT is significantly related to Noise. KS2 Results are significantly related to $L_{A,max}$ (strongest). KS1-Reading & KS2-English are significantly associated to $L_{A,eq}$, $L_{A,max}$, & $L_{A,10}$. KS2-English scores are significantly associated to $L_{A,eq}$, $L_{A,max}$, $L_{A,10}$ & $L_{A,90}$ level. FSM: KS1-Reading & KS2-English are related to external noise. KS2-English are associated to $L_{A,max}$. EAL & SEN: KS2 scores are associated to $L_{A,max}$. KS2-English is related to $L_{A,max}$. [Internal: 2yr vs 6yr] KS1 & KS2 SAT scores are associated to internal noise. In terms of the year group, scores (i.e., except of KS1-Reading) are significantly correlated to noise levels. KS2-English are significantly associated to $L_{A,eq}$ & $L_{A,90}$. Adjustment: KS2-English are significantly associated to internal noise. FSM: $L_{A,90}$ is significantly correlated to test scores (6yr). In terms of locations: all scores are significantly associated to noise levels (occupied/unoccupied classrooms, corridors and foyers). Test scores (occupied) are significantly associated to $L_{A,90}$. KS2-English (occupied) are significantly associated to $L_{A,90}$. KS1-Mathematics (occupied & unoccupied) are significantly correlated to $L_{A,90}$. KS2-English scores are significantly related to all noise levels (corridors & foyers). KS2-English are significantly associated to $L_{A,eq}$ (occupied, corridors,foyers). Full Adjustment: KS2-English scores are significantly associated to $L_{A,90}$ (occupied/corridors/foyers). A 5dB increase in background noise level in occupied conditions is associated to decrease in the required levels of KS1 & KS2.
Van Kempen et al. (2010)† [21] (n=2884, 9-11yrs)	Aircraft Noise (Calculations: Noise Contours) and Road Traffic Noise (Calculations: NSM Methods).	School (Outdoor: Around Airports)	$L_{A,eq,16h}$ (07:00-23:00)	NES Test	Errors for SAT Switch condition are significantly associated to aircraft noise exposure at school. An increase of 10 dBA aircraft noise exposure at school is associated to an increase in errors. Cognitive outcomes are non-associated to aircraft noise exposure at home. Errors for SAT arrow-condition are significantly related to road traffic noise at school. An increase of 10 dBA road traffic noise at school are associated to an increase in errors. Cognitive outcomes are not associated to road traffic noise exposure at home.

†: Cross-sectional study, ∗: case study

Table S5. Well-being dimensions-based studies. %HA refers to high in annoyance percentage.

Article (Participants, Age Range)	Noise Source (Assessment: Method)	Noise Assessment Location (Environment)	Noise Indicators	Health Parameter (Method)	Outcomes
Clark et al. (2013)‡ [9] (n=461, 15.5-17.7yrs)	Aircraft Noise (Calculations: Noise Contours)	Primary and Secondary schools (Outdoor)	$L_{A,eq,16h}$ (07:00-23:00)	Noise Annoyance (ISO Questionnaire)	An increase of 1dB aircraft & cumulative aircraft noise in primary & secondary school is significantly associated to increase in annoyance.
Klatte et al. (2017)† [10] (n=1243, 7-10yrs)	Aircraft Noise (Calculations: Noise Contours)	Schools and homes (Outdoor)	Schools: $L_{A,eq,6h}$ (08-14) Homes: $L_{A,eq,12h}$ (06-18), $L_{A,eq,10h}$ (20-06)	Annoyance (Self-reported assessment), QoL (KINDL-R: Reported (parents) & Self-reported assessment)	An increase in aircraft noise is significantly associated to increase in annoyance responses. An increase of 10dB aircraft noise is associated to an increase in points (4-point rating scale) variables ratings are significantly associated to aircraft noise in QoL. An increase of 10dB aircraft noise is associated to a significant decrease in physical, mental well-being, and well-being at school.
Ali (2013)′ [22] (n=300, 13-15yrs)	Road traffic, and in-classroom noise: (Measurements: B&K 2230)	Schools (Indoor)	$L_{A,eq,20m}$ (4-5 min. measurements)	Annoyance Level (Questionnaire)	School noise levels are associated to %HA Respondents, presenting a non-linear behaviour. Road traffic noise has been considered as the main source of annoyance. 62% of participants reported annoyance, from which its 46.5% reported high annoyance.
Stansfeld (2005)† [11] (n=2844, 9-10yrs)	Road traffic & Aircraft Noise (Calculations and Measurements)	School (Outdoor & Indoor: In testing time)	$L_{A,eq,16h}$ (07:00-23:00)	Annoyance (Adult-based Questionnaire)	Increase in aircraft and road traffic noise is associated to annoyance.
Silva (2016)^{o} [23] (n=213, 8-11yrs)	Ambient Noise (Measurements: CESVA, SC310)	Schools (Outdoor & Indoor: Open school (students inside) & closed school (empty school).	$L_{A,eq,30m}$, $L_{A,95}$	Annoyance (Questionnaire)	Increase in Annoyance (in classroom) is associated to colleagues' voices, corresponding to decrease in activities concentration. Environmental noise $L_{A,eq,30m}$ road traffic noise, and background noise $L_{A,95}$ (inside classroom) is associated to decrease in students with difficulties in hearing the teacher's voice was presented.
Van Kempen (2009)† [24] (n=2844, 9-11yrs)	Aircraft (Calculations: Noise Contour) & Road Traffic Noise (Calculations: CRTN & Others)	School and Home (Outdoor: Around Airports).	$L_{A,eq,16h}$ (07:00-23:00)	Annoyance & Interference in activities (School/Home) (Self-reported questionnaire)	Increase of aircraft noise at school & home is related to severe annoyance. %HA children are associated to increase in NL sample. An increase of 1dB Noise does not differ across countries. Exposure response relationships at home approach exposure response relationships at school. Road traffic noise exposure at school is significantly associated to severe annoyance. Increase in road traffic noise at school is related to increase in annoyance. %HA children are significantly associated to aircraft noise (strong). An increase of 1dB noise do not differ %HA children across countries & noise source.
Haines et al. (2001)† [8] (n=340, 8-11yrs)	Aircraft Noise (Calculations: Noise Contour & Measurements)	School (Outdoor: Selected Schools) & School (Indoor: Classroom)	$L_{A,eq,16h}$	Annoyance: (Questionnaire) Motivation: (Adapted G&S)	Motivation does not differ across groups. Chronic exposure to high aircraft noise levels is significantly associated to annoyance. Annoyance is not related to other noise sources.
Haines et al. (2001)† [13] (n=275, 8-11yrs)	Aircraft Noise (Calculations: Noise Contour & Measurements)	School (Outdoor: Selected Schools) & School (Indoor: Classroom)	$L_{A,eq,16h}$	Annoyance (Seven child adapted standard questions)	High levels of aircraft noise was significantly associated to higher levels of annoyance in the analyses of the eight schools.
Minichilli et al. (2018)† [25] (n=521, 11-17yrs)	Road-Traffic Noise (Measurements: Class 1 SA)	School (Outdoor: School façade, Indoor: Centre of room & 1m from open windows)	$L_{A,eq,ext,30m}$ $L_{A,eq,int,30m}$	Annoyance (Questionnaire)	Industrialized areas are associated to significant increase in mean annoyance index. Perceived noise (younger students) differs significantly across age groups. MAI is marginally related to GNS. Decrease in MAI is associated to increase in acoustic quality (classroom). Increase in GNS is associated to decrease in perceived noise and annoyance. MAI is significantly associated to $L_{eq,ext}$, $L_{eq,int}$ & RT. MAI is marginally associated to STI. Annoyance is not associated to façade & wall insulation. Annoyance at school is significantly related to $L_{eq,ext}$, GNS, $L_{eq,ext}$ & RT. Annoyance at school is marginally associated to STI. Disturbance-fellow students (classroom) is significantly related to $L_{eq,int}$ & $L_{eq,ext}$. Frequency of perceived noise is significantly related to all noise parameters (except of two concerning structural characteristics of classroom). Individual destruction is related to any noise parameter.
Birk et al. (2004)† [26] (n=951, 10yrs)	Road Traffic Noise (Calculations: ECEND 2002/49/CE)	Home (Outdoor: Edge of the City)	L_{den}	Annoyance (Questionnaire Miedema & Oudshoorn model).	"Low" annoyance is significantly associated to road traffic noise (lowest/lower quintile). "Low" annoyance is significantly related to increase of distance to a major road & two types of streets. "High" category is related to other determinants. "Highest" road traffic noise quintile is marginally related to the distance to a major road. "High" category is related to the type of the street of residence "main street" (stronger). Noise annoyance is related to noise level. Annoyance is associated distance to a major road. Noise annoyance is associated to type of residence street. Increase in annoyance is associated to a significant increase in measured noise & type of residence street. Fair agreement: Reported noise annoyance and GIS modelled noise exposure (immediate neighbourhood of the place of residence-L_{den}).
Dockrell et al. (2004)† [27] (n=2063, 6-7/10-11yrs)	Ambient Noise (Measurements: B&K 2236)	School (Outdoor: Most exposed façade)	$L_{A,eq,5m}$, $L_{A,10}$, $L_{A,90}$, $L_{A,max}$, $L_{A,99}$	Annoyance (Questionnaire)	Ratings of annoyance (home/school) are significantly associated to sound sources. A significant increase of annoyance is related to younger children in terms of trains and motorbikes. Younger children annoyance is significantly associated to external noise sources (heard).Older children are aware of external noise sources. School hearing scores are significantly related to $L_{A,99}$. School annoy scores are significantly related to $L_{A,max}$, $L_{A,eq}$, & $L_{A,90}$. Children annoy scores are significantly correlated to $L_{A,max}$. Ambient & maximum levels are associated to factors in reported annoyance.
Lercher et al. (2013)† [5] (n=115, mean 10yrs)	Road-Traffic Noise (Calculations: Modelled Traffic Noise)	Home (Outdoor: Child's Locations)	L_{dn}	Child's Health Related QoL: (KINDL)	Low noise children's areas are significantly associated to increase in HRQoL.

†: Cross-sectional study, o: in-situ study, ′: social survey

Table S6. Mental health effects-based studies.

Article (Participants, Age Range)	Noise Source (Assessment: Method)	Noise Assessment Location (Environment)	Noise Indicators	Health Parameter (Method)	Outcomes
Clark et al. (2013)‡ [9] (n=461, 15.5-17.7yrs)	Aircraft Noise (Calculations: Noise Contours)	Primary and Secondary schools (Outdoor)	$L_{A,eq,16h}$ (07:00-23:00)	Psychological Morbidity (SDQ)	Non-significant associations have been seen with respect to overall difficulties, hyperactivity, conduct scores, and emotional scores.
Tiesler et al. (2017)† [28] (n=872, 10yrs)	Road-Traffic Noise (Calculations: Noise Maps)	Home (Outdoor: Most/least exposed façade)	L_{den}	Psychological Morbidity (SDQ)	Noise exposure (Home) are not associated to overall mental health problems. Increase of noise at most exposed façade is significantly associated to increase in hyperactivity/inattention symptoms. Conduct problems are not related to L_{den}. Noise at least exposed façade is associated to increase in odds, corresponding to borderline or abnormal values on the emotional symptoms. All Road traffic noise variables presented non-significant increase in overall mental health.
Stansfeld et al. (2009)† [29] (n=2844, 9-10yrs)	Road traffic & Aircraft Noise (Calculations and Measurements)	School (Outdoor: School façade)	$L_{A,eq,16h}$ (07:00-23:00)	Mental Health (Questionnaire) Psychological Morbidity (SDQ)	Overall difficulties: NL scores are approximately equal to total parental SDQ scores (5–10y children: UK), with marginal significant difference between UK & SP. Aircraft & road traffic noise exposure are not associate to mental health (total SDQ score). Hyperactivity: NL scores are approximately equal to UK scores, with UK differs from SP. Increase in hyperactivity (full adjusted) is significantly associate to aircraft noise. Significant differences in hyperactivity have been seen across countries, with strongest being in hyperactivity of NL. Conduct: NL scores are approximately equal to UK scores. Increase in SP scores is related to UK & NL scores. Road traffic noise is not related to conduct problems (inverse association/fully adjusted model). Scores presented non-significant differences across countries. Emotional: Scores are not approximately equal to models & both sources. Peer problems: Significant increase in peer scores have been seen in UK. Peer problems are not related to models & both sources. Increase in prosocial behaviour is presented in NL. Prosocial behaviour is not associated to models & both sources. ADHD: Non-significant increase in hyperactivity score has been seen in ADHD group.
Van Kempen et al. (2010)† [4] (n=2884, 9-11yrs)	Aircraft Noise (Calculations: Noise Contours) and Road Traffic Noise (Calculations: NSM Methods).	School (Outdoor: Around Airports)	$L_{A,eq,16h}$ (07:00-23:00)	Perceived-Health (Questionnaire)	Annoyed children by aircraft and road traffic noise at school presented a significant increase in symptoms. Noise exposure at school is not related to self-reported health symptoms. Air and road traffic noise exposure at school is associated to a non-significant increase in symptoms and neurobehavioral functioning.
Hjortebjerg et al. (2016)| [30] (n=46940, 0-7yrs)	Road-Traffic Noise (Calculations: Nordic Method)	Home (Outdoor: Most exposed façade)	L_{den}	Psychological Morbidity (SDQ)	Time-weighted mean exposure from 0-7 years of age is associated to an increase of 10dB road traffic noise, corresponding to a significant increase in abnormal total difficulties scores. Exposure during pregnancy is significantly associated to total difficulties scores and not to abnormal total difficulties scores. For both exposure time windows (adjusting for airport and railway noise) non-significant differences have been seen in terms of road traffic noise. However, significant increases have been seen w.r.t. abnormal scores for total difficulties score or any of the subscales. Adjusting for road traffic and airport noise, non-significant odds for railway noise at birth or at 7 years of age have been extracted. An increase of 10-dB railway noise (at 7 years) is significantly associated to increase in abnormal scores for total difficulties. Hyperactivity/inattention: An increase of 10-dB road traffic noise exposure is significantly, associated to borderline and abnormal scores (adjusted models). LBW children: Road traffic noise is significantly associated to hyperactivity/inattention. An increase of 10dB road traffic noise is not associated to abnormal conduct problem scores. An increase of 10dB road traffic noise is not related to abnormal peer scores. Exposure to road traffic noise (during pregnancy) is related to behavioural problems and remaining outcomes.
Haines et al. (2001)† [8] (n=340, 8-11yrs)	Aircraft Noise (Calculations: Noise Contour & Measurements)	School (Outdoor: Selected Schools) & School (Indoor: Classroom)	$L_{A,eq,16h}$	General symptoms (Standard self-report questions), Psychological Morbidity (SDQ), Anxiety (CMA), Depression (CDI)	Aircraft noise exposure at school is not related to general health, headaches, tiredness & sleeping troubles. Aircraft noise exposure at school is not related to psychological morbidity factors (SDQ completed by parents). Aircraft noise exposure is related to non-significant increase in mean score of anxiety and depression.
Haines et al. (2001)† [13] (n=275, 8-11yrs)	Aircraft Noise (Calculations: Noise Contour & Measurements)	School (Outdoor: Selected Schools) & School (Indoor: Classroom)	$L_{A,eq,16h}$	Lewis' child stress scale.	Aircraft noise was significantly associated with higher levels of perceived stress in the eight and seven schools. Aircraft noise was not associated to the prevalence of stressful life events nor with the total stress score
Weyde et al. (2017)†† [31] (n=1934/1384, 0/3-8yrs)	Road Traffic Noise (Calculations: Nordic Method)	Home (Outdoor: Most exposed façade)	L_{den}	Inattention (Questionnaire-Part of RSDBD).	Pregnancy (road traffic noise) is not related to inattention at age 8 years (full pregnancy sample). Significant increase in inattention for boys is associated to road traffic noise. Significant increase in inattention (for all levels of income) is associated to high noise levels. Road traffic noise exposure (at age 8 years) is related to fractioned inattention score. An increase of 10 L_{den}dB is related to a marginal increase in inattention score. Similar noise estimates were found in five-year road traffic noise exposure.
Lim et al. (2018)† [32] (n=918, 9-14yrs)	Road Traffic Noise (Calculations: Modelled Traffic Noise)	School (Outdoor: External walls)	L_{dn}, Noise Sensitivity	Internalizing problems (CBCL-Anxious/Depressed, Withdrawn/ Depressed, & Somatic Complaints subscales), Externalizing problems (CBCL-Rule-Breaking Behaviour & Aggressive Behavioursubscales).	Noise sensitivity is significantly related to internalizing, externalizing, & total behavioural problems. Noise level are significantly related to total behavioural problems (full adjustment). Noise levels are not related to internalizing and externalizing problems (full adjustment). Internalizing Problems: Noise sensitivity is significantly associated to clinical range of internalizing problems. Internalizing problems are associated to maternal hypertension during pregnancy and preeclampsia. Externalizing problems: Noise sensitivity is significantly related to clinical range of externalizing problems. Total behavioural problems significantly associated to noise level and noise sensitivity (with/without multi-step adjustment).
Crombie et al. (2011)† [33] (n=160, 9-10yrs)	Aircraft Noise (Calculations: Noise Contour) and Road Traffic Noise (Calculations: Modelled)	School (Outdoor: Around airports)	$L_{A,eq,16h}$ (07:00-23:00)	Psychological Morbidity (SDQ)	Aircraft noise is not associated to overall mental health score. Road traffic noise is not related to overall SDQ. Aircraft noise is significantly associated to hyperactivity (after adjustment). Aircraft noise is significantly associate to hyperactivity (after further adjustment). Road traffic noise is not associated to hyperactivity. Aircraft noise is not related to conduct problems. Road traffic noise is significantly associated to conduct problems (after adjustment). Aircraft and road traffic noise are not associated to emotional symptoms.
Belojevic et al. (2012)† [34] (n=311, 7-11yrs)	Aircraft Noise (Measurements: B&K 2250)	Home and Schools (Outdoor: At home streets and in front of schools)	Home: $L_{A,eq,15m}$ (08-10, 14-16, 18-20, 22-00, 00-02) $L_{A,eq,24h}$. School: $L_{A,eq,15m}$ (09-11, 12-14, 15-17)	Children's EF skills (Adapted ADD Questionnaire)	In overall sample, noise levels at school/ home did not provide significant difference in EF. After clustering: Noise levels at home are significantly related to mean EF scores in boys mean EF scores. 24-h noise exposure, road-traffic noise, and gender are significantly associated to children's EF scores (Adjusted model).

†: Cross-sectional study, ‡: follow-up study, ††: longitudinal study, |: cohort study

Table S7. Sleep-based studies.

Article (Participants, Age Range)	Noise Source (Assessment: Method)	Noise Assessment Location (Environment)	Noise Indicators	Health Parameter (Method)	Outcomes
Tiesler et al. (2013)† [28] (n=872, 10yrs)	Road-Traffic Noise (Calculations: Noise Maps)	Home (Outdoor: Most/least exposed façade)	L_n	Difficulties to fall asleep or sleeping through the night (LISAplus cohort data)	Nocturnal noise levels at most exposed façade are not related to sleeping problems. Increase of noise levels at least exposed façade is associated to a significant increase in sleeping problems and difficulties to fall asleep. Significant ORs (Further adjustment) are associated sleeping alone and orientation of the child's room window. L_n is significantly associated to increase in the percentage of sleeping problems & problems to fall asleep (full-adjustment). Presence of any sleeping problems is significantly associate to increase in emotional symptoms. Increase of noise levels at least exposed façade is associated to a non-significant increase in risk for emotional symptoms and decrease in sleeping problems (after adjustment)
Ohrstrom et al. (2006)† [35] (n=160, 9-12yrs)	Road Traffic Noise (Calculations: Nordic Method)	Homes (Outdoor: Most exposed façade)	$L_{A,eq,24h}$	Sleep Evaluation & perceived interference (Questionnaire). Activity mean/ average motility, activity index, sleep latency, sleep minutes & wake episodes/ awakenings (Wrist- actigraphy: Mini-motion-logger actigraphy tri-mode from Ambulatory Monitoring Inc.) Time of going to bed, of getting up, bedroom window open during night, difficulties in falling asleep, time to falling asleep and number of awakenings (Sleep Logs).	$L_{A,eq,24h}$ from < 55 to $> 64dB$ are significantly related to decrease in sleep quality. Mean average sleep quality and alertness (morning) are not associated to the reported sleep quality (in-depth study). Increase in $L_{A,eq,24h}$ is associated to increase in sleepiness (daytime). Feeling alertness in morning is not differ significantly across $L_{A,eq,24h}$ categories. The percentage of waking ups at night do not differ significantly across $L_{A,eq,24h}$ categories. $L_{A,eq,24h} > 64$ & $55 - 59$ dBA are related to non-significant increase in percentages of falling asleep. Non-significant increase in reporting bedroom windows open at night between (seldom/never) in relation to $L_{A,eq,24h}$ highest & lowest group.Inference with sleep $L_{A,eq,24h}$ from < 55 to $60 - 64$dB is related to non-significant increase w.r.t. interference. Interference with sleep (road traffic noise) in "often/always" group is related to non-significant increase in falling sleep. "Never/sometimes" group reported non-significant increase in leaving bedroom windows closed at night (often/always). "often/always" group indicated a significant increase in waking ups (night) and lower sleep quality. "often/always" group (road traffic noise) reported significant decrease in feeling alertness. Sleepiness (daytime) "often/always" group reported significant increase in sleepiness daytime. Children reported significant increase in sleeping quality. Higher noise levels (lowest exposure categories) are associate to marginal decrease in sleeping quality. Children's minutes do not differ with parents' minutes. Children indicated a significant increase of waking up episodes. Children (lowest exposure categories) reported significant decrease of tiredness (morning). Children presented significant increase in time in beds. $L_{A,eq,24h} > 64$dB is related to significant decrease in the percentage of interferences. Children presented significant increase in mean activity. Children indicated significant increase in sleeping latency. Children indicated significant increase in waking-ups episodes. Children presented significant increase in movements (in sleep). Children presented significant increase in sleep minutes.
Weyde et al. (2017)† [36] (n=2665, 6yrs)	Road-Traffic Noise (Calculations: Nordic Method)	Home (Outdoor: Most exposed façade)	L_{en}	Sleep Duration (MoBa 7-year-questionnaire)	Road traffic noise is not related to sleep duration (full adjusted model). Exposure-response is significantly related to girls. An increase of 10dB road traffic noise is associated to increase in Odds (Sleeping 10h or less & less than 10h).

†: Cross-sectional study

3. Assessment of Studies Quality

Table S8. Assessment of the risk of bias in cardiovascular-based studies. RR corresponds to response rate, BR to bias rate and OBR to overall risk of bias

Study	Participation			Assessment Methodology		Exposure Assessment			Confounding Factors		OBR
	RR	Inclusion/Exclusion Criteria	BR	Method	BR	Definition	Measurements	BR	Included in Analysis	BR	
Wallas et al. (2019) [1]	63.5%	Family planned to move within 1 year of the study start, insufficient knowledge of the Swedish language, the family had a seriously ill child, or an older sister or brother was already included in the study (Excluded)	Low	Clinical	Low	L_{den}	Calculations: Most exposed façade	Low	Sex, age, height, municipality at birth, duration of exclusive breastfeeding, maternal education, maternal BMI in pregnancy, maternal smoking during pregnancy, physical activity and nicotine use at 16 years of age.	Low	Low
Babisch et al. (2009) [2]	52.6%	Living in Germany with respect to gender, community size, and region (Included)	High	Clinical	Low	$L_{A,eq}$	Measurements of 15 minutes in front of children open window between 08:00-21:30, depending on the time of the visit.	Low	Age, gender, area, socio-economic status, migrant status, agglomeration size, height, weight and physical activity.	Low	High
Belojevic et al. (2008) [3]	62.8%	Three or more years living on the present address and orientation of a child's bedroom towards the streets. The exclusion criterion for the sample was the presence of chronic diseases affecting arterial blood pressure	Low	Clinical	Low	$L_{A,eq}$	Measured in two night intervals in front of children's residences: between 10 p.m. & 12 a.m. and between 12 a.m. & 1.30 a.m. In front of each kindergarten noise measurements were performed in two daily periods (9a.m.–10.30a.m. & 1.30 p.m.–3 p.m.). Time interval of each measurement was 15 min.	Low	N/A	High	High
Van Kempen et al. (2010) [4]	89%	Noise exposure, socioeconomic status, ethnicity	Low	Clinical	Low	$L_{A,eq}$	Measure and calculated for periods higher or equal to 3 months.	Low	Noise at school, age, sex, coutry, SES, outcome-specific variable, annoyance, and annoyance noise at school	Low	Low
Lercher et al. (2013) [5]	64%	Noise exposure and educational background of mothers	Low	Clinical	Low	L_{dn}	Calculations and validation measurements	Low	BMI, sex, house type, education, cortisol, area quiet, sound*gestation, sound*cortisol	Low	Low

Table S9. Assessment of the risk of bias in neuroendocrine-based studies. For explanation of symbols and notations, see caption of Table S8.

Study	Participation			Assessment Methodology		Exposure Assessment			Confounding Factors		OBR
	RR	Inclusion/Exclusion Criteria	BR	Method	BR	Definition	Measurements	BR	Included in Analysis	BR	
Wallas et al. (2018) [6]	50%	Samples contained too little material for analysis and individuals without information on relevant covariates.	Low	Clinical	Low	L_{den}	Calculations: Most exposed façade	Low	Age, sex, rhinitis, eczema and sampling season.	Low	Low
Canturia et al. (2018) [7]	80.5%	Exclusion criteria: delivery < 35 weeks gestational age and problems during the extraction of urine samples and/or analysis.	Low	Clinical	Low	L_{den}	Calculations	Low	Sex, gestational age at birth and weight at the time of urine collection, maternal smoking during pregnancy, delivery mode, occurrence of vaginal infection, maternal education, existence of older siblings, season at birth and NO2 exposure during pregnancy.	Low	Low
Haines et al. (2001) [8]	77%	Age of the children, sound level at the school from non-aircraft sources, the extent of existing noise protection in the schools, socio-economic group distribution and unemployment rate, and proportion of ethnic groups in area based on electoral ward analysis.	Low	Clinical	Low	$L_{A,eq}$	Calculations and measurements during cognitive test	Low	Age and time of sample collection and not main language and deprivation.	Low	Low
Lercher et al. (2013) [5]	64%	Noise exposure and educational background of mothers	Low	Clinical	Low	L_{dn}	Calculations and validation measurements	Low	BMI, sex, house type, education, cortisol, area quiet, sound*gestation, sound*cortisol	Low	Low

Table S10. Assessment of the risk of bias in cognitive development-based studies. For explanation of symbols and notations, see caption of Table S8.

Study	Participation			Assessment Methodology		Exposure Assessment			Confounding Factors		OBR
	RR	Inclusion/Exclusion Criteria	BR	Method	BR	Definition	Measurements	BR	Included in Analysis	BR	
Clark et al. (2013) [9]	45.4%	Participants left the school, absent, attended special needs school or schools with were excluded.	Low	Suffolk Reading, Scale 2, Level 2 at baseline & Level 3 at follow-up	Low	L_{den}	Calculations & measurements during the test	Low	Age, gender, parental employment, crowding in the home, home ownership, mother's education, long standing illness, main parental support, classroom glazing, and road noise at primary school.	Low	Low
Klatte et al. (2017) [10]	N/A	Schools in which the headmasters reported high annoyance due to road-traffic, railroad traffic, building sites, industry, or other noise sources were exclude	Unclear	Standardized reading comprehension test for primary school children instructed in German	Low	$L_{A,eq}$	Calculations	Low	Age, gender, non-verbal abilities, SES, migration background, number of children's books at home, and German language proficiency, percentage of children with a migration background in the class, mean SES, class size, and parental involvement. In case of reading scores as outcome variables, story comprehension, rapid access to phonological word representations, and phonological awareness were also included as Level 1 variable	Low	High
Stansfeld et al. (2005) [11]	89%	Exclusion of non-state schools in the UK and Spain, excluded schools at which noise surveys indicated either the presence of a dominant noise other than aircraft or road traffic noise, or at which insulation against noise was above a certain threshold.	Low	Adapted TPT for classroom use, Task from CMS, modified version of search & memory task, SRS, CITO	Low	$L_{A,eq}$	Calculations and measurements	Low	Age, sex, country, noise, socioeconomic status and mother's education, children's longstanding illness, main language spoken at home, parental support for schoolwork, and the type of glazing in the windows of the child's classroom	Low	Low
Seabi et al. (2015) [12]	Attrib. $< 20\%$	Some learners in Grade 8 (i.e., new schools) was not granted by some school principals, relocation and learners absent from school (weather conditions).	High	Questionnaire: Suffolk Reading Scale Level 2	Low	$L_{A,eq}$ 2.5h during test	Measurements	Low	Gender, language, deprivation	Low	High
Haines et al. (2001) [8]	77%	Age of the children, sound level at the school from non-aircraft sources, the extent of existing noise protection in the schools, socio-economic group distribution and unemployment rate, and proportion of ethnic groups in area based on electoral ward analysis.	Low	Reading material, serial digit recall, SRSL2	Low	$L_{A,eq}$	Calculations and measurements during cognitive test	Low	Age, deprivation and main language spoken.	Low	Low
Haines et al. (2001) [13]	81%	School children from noisy and quiet areas, around airport.	Low	Child adapted standard questions	Low	$L_{A,eq}$	Calculations and measurements during cognitive test	Low	Age, deprivation and main language spoken.	Low	Low
Clark et al. (2006) [14]	89%	Socioeconomic status, main language spoken at home, recent immigrants who do not speak the main language of the country	Low	UK: SRSL-2, NL: CITO, SP: ECL-2	Low	$L_{A,eq}$	Calculations	Low	Age, gender, country, mother's education, employment status, crowding, home ownership, long-standing illness, main language spoken at home, parental support, classroom glazing, and road traffic noise exposure.	Low	Low
Matsui et al. (2004) [15]	83% HN 81% LN	Exclusion of sample from the low noise schools	Low	TEA-ch, Adapted CMS, SRS	Low	$L_{A,eq}$	Calculations	Low	Sex, age, deprivation score, mother's education level, schools and spoken language at home, version of Suffolk reading test.	Low	Low
Matheson et al. (2010) [16]	89%	Socioeconomic position, number of pupils eligible for free school meals and main language spoken at home beginning with those schools exposed to the highest levels of aircraft noise. Schools were then excluded if they had a dominant noise source other than aircraft or road traffic noise. In the UK & Spain, two classes were selected in each school as far as possible and in the Netherlands one class was selected in each school.	Low	Children's Memory Scale, Shield & Dockrell test)	Low	$L_{A,eq}$	Calculations	Low	Noise exposure, either aircraft or road traffic noise, classroom glazing, i.e., the type of windows in the child's classroom, age, sex, country, mother's educational attainment, socioeconomic status, crowding and home ownership, LSI, main language spoken at home and parental support for school work and the other noise exposure variable, parental report of the child having dyslexia and acute noise during testing.	Low	Low

Table S11. Assessment of the risk of bias in cognitive performance-based studies. For explanation of symbols and notations, see caption of Table S8.

Study	Participation			Assessment Methodology		Exposure Assessment			Confounding Factors		OBR
	RR	Inclusion/Exclusion Criteria	BR	Method	BR	Definition	Measurements	BR	Included in Analysis	BR	
Xie et al. (2011) [17]	N/A	Location	High	Achievements, Attainment Table KS4,CVA scores	Low	$L_{A,eq}$	Calculations	Low	N/A	High	High
Pujol et al. (2014) [18]	77.4%	Change in address and hearing impairment	Low	NAS Test	Low	L_{den}, $L_{A,eq}$ L_d, L_e, L_n	Calculations	Low	Socioeconomic status, employment, parental education level, age, sex, main language spoken at home, reading (leisure activity)	Low	Low
Van Kempen et al. (2010) [4]	89%	Noise exposure, socio-economic status, ethnicity	Low	NES Test	Low	$L_{A,eq}$	Calculations and measurements	Low	NO2, PM10, age, gender, main language spoken at home, long-standing illness, parental support for school work, school glazing, socioeconomic status, crowding, home ownership, parental employment, mother's education	Low	Low
Haines et al. (2002) [19]	N/A	Classification of school in noise	Unclear	SATs: KS2	Low	$L_{A,eq}$	Calculations	Low	Sex, year of testing and type of school, free school meals.	Low	High
Shield et al. (2008) [20]	N/A	School location	Unclear	SAT KS1/KS2	Low	$L_{A,eq}$, $L_{A,90}$ $L_{A,10}$	Measurements 5 minn during test	Low	Free meal, English as an additional language, special needs children	Low	High
Van Kempen et al. (2010) [21]	89%	Schools around airport	Low	HECT, SDST, TPT SRTT, SAT, CMS SMT DMST, CRIE	Low	$L_{A,eq}$	Calculations	Low	Age, gender, main language spoken at home, long standing illness, parental support for school work, school glazing, indicators for socioeconomic status, and other noise source.	Low	Low

Table S12. Assessment of the risk of bias in well-being dimensions-based studies. For explanation of symbols and notations, see caption of Table S8.

Study	Participation			Assessment Methodology		Exposure Assessment			Confounding Factors		OBR
	RR	Inclusion/Exclusion Criteria	BR	Method	BR	Definition	Measurements	BR	Included in Analysis	BR	
Clark et al. (2013) [9]	45.4%	Participants left the school, absent, attended special needs school or schools with were excluded.	Low	ISO Questionnaire	Low	L_{den}	Calculations & measurements during the test	Low	Age, gender, parental employment, crowding in the home, home ownership, mother's education, long standing illness, main parental support, classroom glazing, and road noise at primary school.	Low	Low
Klatte et al. (2017) [10]	N/A	Schools in which the headmasters reported high annoyance due to road-traffic, railroad traffic, building sites, industry, or other noise sources were exclude	Unclear	Standardized reading comprehension test for primary school children instructed in German	Low	$L_{A,eq}$	Calculations	Low	Age, gender, SES, road traffic noise, rail traffic noise at home, classroom insulation	Low	High
Ali (2013) [22]	62.3%	Schools were chosen to have a wide geographical distribution	Low	Non-Standardized Questionnaire	High	$L_{A,eq}$	Measurements 4-5min	High	N/A	High	High
Stansfeld et al. (2005) [11]	89%	Exclusion of non-state schools in the UK and Spain, excluded schools at which noise surveys indicated either the presence of a dominant noise other than aircraft or road traffic noise, or at which insulation against noise was above a certain threshold.	Low	Standardized Questionnaire	Low	$L_{A,eq}$	Calculations and measurements	Low	Age, sex, and country	Low	Low
Silva et al. (2016) [23]	51.2%	N/A	High	Non-Standardized Questionnaire	High	$L_{A,eq}$	Measurements, 30min Non-specified period	High	N/A	High	High
Van Kempen et al. (2009) [24]	89%	Primary schools close to airports	Low	Standardized Questionnaire	Low	$L_{A,eq}$	Calculations	Low	Age years , sex, ethnicity, school glazing, glazing at home, length of school enrolment, indicators of SES.	Low	Low
Haines et al. (2001) [8]	77%	Age of the children, sound level at the school from non-aircraft sources, the extent of existing noise protection in the schools, socioeconomic group distribution and unemployment rate, proportion of ethnic groups in area based on electoral ward analysis.	Low	Non-Standardized Questionnaire	High	$L_{A,eq}$	Measurements, and Calculations	Low	Age, deprivation and main language spoken	Low	High
Haines et al. (2001) [13]	81%	Age of the children, sound level at the school from non-aircraft sources, the extent of existing noise protection in the schools, socioeconomic group distribution and unemployment rate, proportion of ethnic groups in area based on electoral ward analysis.	Low	Standardized Questionnaire	Low	SEL	Measurements, and Calculations	Low	Age, deprivation and main language spoken	Low	Low
Minichilli et al. (2018) [25]	89%	Italian areas distributed along the national territory, with different socio-economic and environmental pressures	Low	Non-Standardized Questionnaire	High	$L_{A,eq}$	Measurements Out/In	Low	Area, age group, gender	Low	High
Birk et al. (2011) [26]	89%	Children born in Munich and live in the city of Munich at the age of 10 years	Low	Non-Standardized Questionnaire	High	L_{den}	Calculations	Low	Exposure, Distance to main road, type of major road, family income, parental education	Low	High
Dockrell et al. (2004) [27]	89%	SES among London primary schools and a range of primary school environment	Low	Non-Standardized Questionnaire	High	$L_{A,eq,5m}$, $L_{A,10}$, $L_{A,90}$,, $L_{A,max}$, $L_{A,99}$	Measurements	Low	N/A	High	High
Lercher et al. (2013) [5]	64%	Noise exposure, educational status of their mothers	Low	Non-Standardized Questionnaire	High	L_{dn}	Measurements and Claculations	Low	N/A	High	High

Table S13. Assessment of the risk of bias in mental health effects-based studies. For explanation of symbols and notations, see caption of Table S8.

Study	Participation			Assessment Methodology		Exposure Assessment			Confounding Factors		OBR
	RR	Inclusion/Exclusion Criteria	BR	Method	BR	Definition	Measurements	BR	Included in Analysis	BR	
Clark et al. (2013) [9]	45.4%	Participants left the school, absent, attended special needs school or schools with were excluded.	Low	SDQ	Low	L_{den}	Calculations & measurements during the test	Low	Age, gender, parental employment, crowding in the home, home ownership, mother's education, long standing illness, main parental support, classroom glazing, and road noise at primary school.	Low	Low
Tiesler et al. (2013) [28]	N/A	Participation at the 10-year follow-up, the availability of noise exposure data (home address in the city of Munich) and information on behavioural problems. Excluded children who were living for less than 1 year at their current place of residence.	Unclear	SDQ	Low	L_{den}	Calculations	Low	Sex, child's age, parental educational level, mother's age at birth, television/computer usage and single parent status.	Low	High
Stansfeld et al. (2005) [11]	89%	Exclusion of non-state schools in the UK and Spain, excluded schools at which noise surveys indicated either the presence of a dominant noise other than aircraft/road traffic noise, or at which insulation against noise was above a certain threshold.	Low	SDQ	Low	$L_{A,eq}$	Calculations and Measurements	Low	Age, gender, country, mother's education, employment status, crowding, homeownership, long-standing illness, main language spoken at home, parental support, classroom glazing and other noise exposure.	Low	Low
Van Kempen et al. (2010) [4]	89%	Noise exposure, SES, ethnicity	Low	Self-reported Symptoms	Low	$L_{A,eq}$	Calculations and Measurements	Low	Noise at school, age, sex, country, SES, outcome-specific variable, annoyance, annoyance noise at school	Low	Low
Hjortebjerg et al. (2016) [30]	N/A	Children based on Danish National Birth Cohort	Unclear	ICD-10, SDQ	Low	L_{den}	Calculations	Low	Sex, age at SDQ, gestational age, birthweight, maternal age at delivery, parity, educational level, disposable income, smoking and alcohol consumption during 1st trimester, railway and airport noise at birth (for exposure during pregnancy) and at 7 years of age, and self-reported maternal mental health problems during 1st trimester (yes/no).	Low	High
Haines et al. (2001) [8]	77%	Age of the children, sound level at schools from non-aircraft sources, extent of existing noise protection in schools, SES group distribution & unemployment rate, proportion of ethnic groups in area.	Low	CAS, SDQ CMA, CDI	Low	$L_{A,eq}$	Measurements, and Calculations	Low	Age, deprivation and main language spoken	Low	Low
Haines et al. (2001) [13]	81%	ge of the children, sound level at schools from non-aircraft sources, extent of existing noise protection in schools, SES group distribution & unemployment rate, proportion of ethnic groups in area.	Low	CMAS, CDI	Low	$L_{A,eq}$	Measurements, and Calculations	Low	Age, deprivation and main language spoken	Low	Low
Weyde et al. (2017) [31]	40.6%	Children born from 2004 to 2007	Low	RSBD-item	Low	$L_{A,eq}$	Calculations	Low	Road traffic noise, age, gender, household income, maternal education, urbanity, ethnicity, maternal alcohol consumption during pregnancy, maternal smoking during pregnancy, low birth weight and prematurity	Low	Low
Lim et al. (2018) [32]	N/A	Children born from 2004 to 2007	Unclear	Behavioural Checklist	Low	L_{dn}	Calculations	Low	Age, sex, income, premature birth, maternal age at birth, maternal disease during pregnancy (hypertension, preeclampsia), passive smoking, mental disorders (ADHD, tic disorder, conduct disorder)	Low	High
Crombie et al. (2011) [33]	89%	Schools around airport	Low	SDQ	Low	$L_{A,eq}$	Calculations and Measurements	Low	Country, age, gender, employment status, crowding, home ownership, mother's educational achievement, long-standing illness, main language spoken at home, parental support for schoolwork and classroom glazing type	Low	Low
Belojevic et al. (2012) [34]	N/A	Public schools in central Belgrade between September 2008 & June 2009	Unclear	Adapted AAD Questionnaire	Low	$L_{A,eq}$	No Continuous Measurements	High	Gender, socioeconomic status, equivalent noise level at home Gender∗$L_{A,eq_2}4$.	Low	High

Table S14. Assessment of the risk of bias in sleep-based studies. For explanation of symbols and notations, see caption of Table S8.

Study	Participation			Assessment Methodology		Exposure Methodology			Confounding Factors		OBR
	RR	Inclusion/Exclusion Criteria	BR	Method	BR	Definition	Measurements	BR	Included in Analysis	BR	
Tiesler et al. (2013) [28]	N/A	Inclusion criteria: participation at the 10-year follow-up, the availability of noise exposure data (home address in the city of Munich) and information on behavioural problems. Additionally, children who were living for less than 1 year at their current place of residence were excluded.	Unclear	Questionnaire	High	L_n	Calculations	Low	Sex, child's age, parental educational level, mother's age at birth, television/computer usage & single parent status.	Low	High
Ohrstrom et al. (2006) [35]	N/A	Noise exposure-based selections and children with normal hearing	Unclear	Questionnaire and Actigraphy	Low	$L_{A,eq,24h}$	Calculations	Low	N/A	High	High
Weyde et al. (2017) [36]	50%	Lived less than 180 days at the present address	High	Sleep Duration (MoBa 7-year-questionnaire)	High	L_{dn}	Calculations	Low	Age, gender, income, season, urbanity, mother's education, ethnicity, siblings in child's household, type of residential building, rail traffic	Low	High

References

1. Wallas, A.E.; Eriksson, C.; Bonamy, A.K.E.; Gruzieva, O.; Kull, I.; Ögren, M.; Pyko, A.; Sjöström, M.; Pershagen, G. Traffic noise and other determinants of blood pressure in adolescence. *Int. J. Hyg. Environ. Health* **2019**, *222*, 824–830.
2. Babisch, W.; Neuhauser, H.; Thamm, M.; Seiwert, M. Blood pressure of 8–14 year old children in relation to traffic noise at home—Results of the German Environmental Survey for Children (GerES IV). *Sci. Total Environ.* **2009**, *407*, 5839–5843.
3. Belojevic, G.; Jakovljevic, B.; Stojanov, V.; Paunovic, K.; Ilic, J. Urban road-traffic noise and blood pressure and heart rate in preschool children. *Environ. Int.* **2008**, *34*, 226–231.
4. Van Kempen, E.; van Kamp, I.; Nilsson, M.; Lammers, J.; Emmen, H.; Clark, C.; Stansfeld, S. The role of annoyance in the relation between transportation noise and children's health and cognition. *J. Acoust. Soc. Am.* **2010**, *128*, 2817–2828.
5. Lercher, P.; Evans, G.W.; Widmann, U. The ecological context of soundscapes for children's blood pressure. *J. Acoust. Soc. Am.* **2013**, *134*, 773–781.
6. Wallas, A.; Eriksson, C.; Gruzieva, O.; Lind, T.; Pyko, A.; Sjöström, M.; Ögren, M.; Pershagen, G. Road traffic noise and determinants of saliva cortisol levels among adolescents. *Int. J. Hyg. Environ. Health* **2018**, *221*, 276–282.
7. Cantuaria, M.L.; Usemann, J.; Proietti, E.; Blanes-Vidal, V.; Dick, B.; Flück, C.E.; Rüedi, S.; Héritier, H.; Wunderli, J.M.; Latzin, P.; et al. Glucocorticoid metabolites in newborns: A marker for traffic noise related stress? *Environ. Int.* **2018**, *117*, 319–326.
8. Haines, N.; Stansfeld, S.; Job, R.; Berglund, B.; Head, J. Chronic aircraft noise exposure, stress responses, mental health and cognitive performance in school children. *Psychol. Med.* **2001**, *31*, 265–277.
9. Clark, C.; Head, J.; Stansfeld, S.A. Longitudinal effects of aircraft noise exposure on children's health and cognition: A six-year follow-up of the UK RANCH cohort. *J. Environ. Psychol.* **2013**, *35*, 1–9.
10. Klatte, M.; Spilski, J.; Mayerl, J.; Möhler, U.; Lachmann, T.; Bergström, K. Effects of Aircraft Noise on Reading and Quality of Life in Primary School Children in Germany: Results From the NORAH Study. *Environ. Behav.* **2017**, *49*, 390–424.
11. Stansfeld, S.; Berglund, B.; Clark, C.; Lopez-Barrio, I.; Fischer, P.; Öhrström, E.; Haines, M.; Head, J.; Hygge, S.; van Kamp, I.; et al. Aircraft and road traffic noise and children's cognition and health: A cross-national study. *Lancet* **2005**, *365*, 1942–1949.
12. Seabi, J.; Cockcroft, K.; Goldschagg, P.; Greyling, M. A prospective follow-up study of the effects of chronic aircraft noise exposure on learners' reading comprehension in South Africa. *J. Expo. Sci. Environ. Epidemiol.* **2015**, *25*, 84–88.
13. Haines, M.; Stansfeld, S.; Soames Job, R.; Berglund, B.; Head, J. A follow-up study of effects of chronic aircraft noise exposure on child stress responses and cognition. *Int. J. Epidemiol.* **2001**, *30*, 839–845.
14. Clark, C.; Martin, R.; Kempen, E.V.; Alfred, T.; Head, J.; Davies, H.W.; Haines, M.M.; Barrio, I.L.; Matheson, M.; Stansfeld, S.A. Exposure-effect relations between aircraft and road traffic noise exposure at school and reading comprehension: The RANCH project. *Am. J. Epidemiol.* **2006**, *163*, 27–37.
15. Matsui, T.; Stansfeld, S.; Haines, M.; Head, J. Children's Cognition and Aircraft Noise Exposure at Home—The West London Schools Study. *Noise Health* **2004**, *7*, 49–58.
16. Matheson, M.; Clark, C.; Martin, R.; van Kempen, I.; Haines, M.; Lopez-Bario, I.; Hygge, S.; Stansfeld, S. The Effects of Road Traffic Noise Exposure on children's Episodic Memory: The RANCH Project. *Noise Health* **2010**, *12*, 244–254.
17. Xie, H.; Kang, J.; Tompsett, R. The impacts of environmental noise on the academic achievements of secondary school students in Greater London. *Appl. Acoust.* **2011**, *72*, 551–555.
18. Pujol, S.; Levain, J.P.; Houot, H.; Petit, R.; Berthillier, M.; Defrance, J.; Lardies, J.; Masselot, C.; Mauny, F. Association between ambient noise exposure and school performance of children living in an urban area: A cross-sectional population-based study. *J. Urban Health* **2014**, *91*, 256–271.
19. Haines, M.M.; Stansfeld, S.A.; Head, J.; Job, R.F. Multilevel modelling of aircraft noise on performance tests in schools around Heathrow Airport London. *J. Epidemiol. Community Health* **2002**, *56*, 139–144.
20. Shield, B.M.; Dockrell, J.E. The effects of environmental and classroom noise on the academic attainments of primary school children. *J. Acoust. Soc. Am.* **2008**, *123*, 133–144.
21. Van Kempen, E.; van Kamp, I.; Lebret, E.; Lammers, J.; Emmen, H.; Stansfeld, S. Neurobehavioral effects of transportation noise in primary schoolchildren: A cross-sectional study. *Environ. Health* **2010**, *9*, 25.
22. Ali, S.A.A. Study effects of school noise on learning achievement and annoyance in Assiut city, Egypt. *Appl. Acoust.* **2013**, *74*, 602–606.
23. Silva, L.T.; Oliveira, I.S.; Silva, J.F. The impact of urban noise on primary schools. Perceptive evaluation and objective assessment. *Appl. Acoust.* **2016**, *106*, 2–9.
24. van Kempen, E.E.; van Kamp, I.; Stellato, R.K.; Lopez-Barrio, I.; Haines, M.M.; Nilsson, M.E.; Clark, C.; Houthuijs, D.; Brunekreef, B.; Berglund, B.; et al. Children's annoyance reactions to aircraft and road traffic noise. *J. Acoust. Soc. Am.* **2009**, *125*, 895–904.
25. Minichilli, F.; Gorini, F.; Ascari, E.; Bianchi, F.; Coi, A.; Fredianelli, L.; Licitra, G.; Manzoli, F.; Mezzasalma, L.; Cori, L. Annoyance judgment and measurements of environmental noise: A focus on italian secondary schools. *Int. J. Environ. Res. Public Health* **2018**, *15*, 208.
26. Birk, M.; Ivina, O.; Klot, S.V.; Babisch, W.; Heinrich, J. Road traffic noise: Self-reported noise annoyance versus GIS modelled road traffic noise exposure. *J. Environ. Monit.* **2011**, *13*, 3237–3245.
27. Dockrell, J.E.; Shield, B. Children's perceptions of their acoustic environment at school and at home. *J. Acoust. Soc. Am.* **2004**, *115*, 2964–2973.

28. Tiesler, C.M.; Birk, M.; Thiering, E.; Kohlböck, G.; Koletzko, S.; Bauer, C.P.; Berdel, D.; Berg, A.V.; Babisch, W.; Heinrich, J.; et al. Exposure to road traffic noise and children's behavioural problems and sleep disturbance: Results from the GINIplus and LISAplus studies. *Environ. Res.* **2013**, *123*, 1–8.
29. Stansfeld, S.A.; Clark, C.; Cameron, R.M.; Alfred, T.; Head, J.; Haines, M.M.; van Kamp, I.; van Kempen, E.; Lopez-Barrio, I. Aircraft and road traffic noise exposure and children's mental health. *J. Environ. Psychol.* **2009**, *29*, 203–207.
30. Hjortebjerg, D.; Andersen, A.M.N.; Christensen, J.S.; Ketzel, M.; Raaschou-Nielsen, O.; Sunyer, J.; Julvez, J.; Forns, J.; Sørensen, M. Exposure to road traffic noise and behavioral problems in 7-year-old children: A cohort study. *Environ. Health Perspect.* **2016**, *124*, 228–234.
31. Weyde, K.V.; Krog, N.H.; Oftedal, B.; Evandt, J.; Magnus, P.; Øverland, S.; Clark, C.; Stansfeld, S.; Aasvang, G.M. Nocturnal road traffic noise exposure and children's sleep duration and sleep problems. *Int. J. Environ. Res. Public Health* **2017**, *14*, 491.
32. Lim, J.; Kweon, K.; Kim, H.W.; Cho, S.W.; Park, J.; Sim, C.S. Negative impact of noise and noise sensitivity on mental health in childhood. *Noise Health* **2018**, *20*, 199–211.
33. Crombie, R.; Clark, C.; Stansfeld, S.A. Environmental noise exposure, early biological risk and mental health in nine to ten year old children: A cross-sectional field study. *Environ. Health* **2011**, *10*, 39.
34. Belojevic, G.; Evans, G.W.; Paunovic, K.; Jakovljevic, B. Traffic noise and executive functioning in urban primary school children: The moderating role of gender. *J. Environ. Psychol.* **2012**, *32*, 337–341.
35. Öhrström, E.; Hadzibajramovic, E.; Holmes, M.; Svensson, H. Effects of road traffic noise on sleep: Studies on children and adults. *J. Environ. Psychol.* **2006**, *26*, 116–126.
36. Weyde, K.V.; Krog, N.H.; Oftedal, B.; Magnus, P.; Øverland, S.; Stansfeld, S.; Nieuwenhuijsen, M.J.; Vrijheid, M.; Pascual, M.D.C.; Aasvang, G.M. Road traffic noise and children's inattention. *Environ. Health* **2017**, *16*, 127.
